# Effects of comprehensive rehabilitation training in combination with multi-mode analgesia on body function recovery after tumor-type knee replacement

**DOI:** 10.12669/pjms.325.10515

**Published:** 2016

**Authors:** Junjuan Zhang, Yahan Wang, Guangyu Yang, Jing Liu, Junjie Wang

**Affiliations:** 1 Junjuan Zhang, Henan Provincial People’s Hospital/Zhengzhou University People’s Hospital, Henan, 450003, China; 2 Yahan Wang, Henan Provincial People’s Hospital/Zhengzhou University People’s Hospital, Henan, 450003, China; 3 Guangyu Yang, Henan Provincial People’s Hospital/Zhengzhou University People’s Hospital, Henan, 450003, China; 4 Jing Liu, Henan Provincial People’s Hospital/Zhengzhou University People’s Hospital, Henan, 450003, China; 5 Junjie Wang, Henan Provincial People’s Hospital/Zhengzhou University People’s Hospital, Henan, 450003, China

**Keywords:** Knees, Multimodal analgesia, Functional training

## Abstract

**Objective:**

To discuss the significance of comprehensive rehabilitation training combined with multimodal analgesia (MMA) for the early knee function recovery of patients with knee bone tumor who underwent prosthesis replacement operation.

**Methods:**

Sixty patients with knee bone tumor who underwent prosthesis replacement operation were selected and randomly divided into two groups according to rehabilitation training and postoperative analgesic methods, namely, observation group and control group, 30 cases in each group. The control group was treated with symptomatic treatment (drugs were given based on pain before and after surgery) and continuous passive motion (CPM) functional training, while the observation group was treated with comprehensive rehabilitation training combined with MMA. The compliance of patients in the two groups was compared and the first-time off-bed activity time was recorded. Recovery conditions of wounds were observed, and recovery conditions of limb functions after operations were evaluated.

**Results:**

The compliance of patients in the observation group was significantly higher than that in the control group, and the difference was statistically significant (P<0.05). The first-time off-bed activity time of patients of the observation group was earlier than that of the control group. The wound recovery condition of the observation group 7 days after operation was better compared to that of the control group, and the difference between two groups two weeks after operation was not statistically significant. The Hospital for special surgery knee (HSS) score and evaluation result of range of motion (ROM) of knees of the observation group were both better than those of the control group in different periods after operation, and the differences were statistically significant (P<0.05).

**Conclusion:**

Comprehensive rehabilitation training combined with MMA can improve the compliance of patients and help patients off bed earlier, and remarkably promote the early recovery of knee functions; hence it deserves to be promoted clinically.

## INTRODUCTION

As the biggest joint of human body, knee joint plays a vital function. The deficiency of knee functions will have serious impacts on the life quality of patients, and knees are sites with high risks of bone tumors, especially malignant bone tumors.^1^ The morbidity of bone tumor is 2% to 3% that of whole body tumors and tends to increase, which greatly threatens the health and safety of patients.^2^ Which treatment to choose for damages that tumor does to knees and how to maximize recovery effects of knee functions under the premise that treatment effects of tumors can be guaranteed have always puzzled orthopedists.

In recent years, with the extensive use of chemotherapy and artificially customized tumorous knee replacement operation, knee prosthesis replacement has been a main rebuilding method of limb salvage treatment for knee peripheral tumors.^3^ Knee prosthesis is featured by early weight bearing, simple operation and satisfactory recovery effect.^4^,^5^ Wunder et al. did a comparative research on 11 cases of irradiated allogeneic bone transplantation and 64 cases of knee joint replacement and found the success rate of prosthesis replacement in the treatment of knee peripheral bone tumors, the relief of operative complications and function recovery were better compared to the rebuilding of knees using allogeneic bones.^6^ Though therapeutic effects of operative treatments for this kind of patients are significant, it may easily cause venous return of affected limbs and the swell and phlebothrombosis if knees are fixed for a long times after tumor bone resection in combination with knee joint replacement is performed on knees with bone tumors. Long-term inaction of knees may also lead to the adhesion, stiffness, pains and other complications of knee joints.^7^,^8^ Therefore, good functional training and rehabilitation nursing for patients treated with tumorous knee replacement operations are essential for improving the life quality of patients, reducing the occurrence of complications and achieving best efficacy.^9^

Though rehabilitation training after Total Knee Arthroplasty (TKA) has been reported more frequently and the studying methods have also been improved gradually in China, studies on systemic rehabilitation in perioperative period of TKA are few. Because of that, this study investigated the effects of comprehensive rehabilitation training combined with multimodal analgesia (MMA) on early functional recovery in perioperative period of TKA.

## METHODS

Sixty patients who suffered from bone tumor on knees and underwent prosthesis replacement operation from Apr. 2013 to Apr. 2015 in Henan Provincial People’s Hospital, Henan, China were selected. This study has been approved by the medical ethics committee of Henan Provincial People’s Hospital and patients signed informed consent. All cases were selected according to inclusion and exclusion criteria below.

### Inclusion criteria

Bone tumors of distal femoral or proximal tibia were evaluated as stage I or II according to Enneking staging; there were no important nerves and blood vessels being invaded, and enough skins and soft tissues were available to cover prosthesis; it was confirmed as malignant tumors or level II or III giant cell tumor of bone by puncture or biopsy; primary sites of tumors were around one side of knees, and no other sites had tumors or tumor metastasis had not happened.

### Exclusion criteria

Patients with severe mental diseases that could not cooperate treatment or who disagreed treatment, patients whose femoral nerves part were infected or had wounds (not appropriate to undergo femoral nerve block) and patients who might be allergic to drugs. Patients were randomly divided into two groups, namely, observation group and control group, according to different postoperative rehabilitation training modes and analgesic methods. The observation group was treated with comprehensive rehabilitation training combined with MMA, among which, 18 cases were males and 12 cases were females, aged from 16 to 32 years (average (25.3±6.4) years). In the observation group, there were 14 cases of distal femoral osteosarcoma, 6 cases of giant cell tumor of bone, one case of Ewing’s sarcoma, two cases of chondrosarcoma, 7 cases of proximal tibia osteosarcoma, and three cases of giant cell tumor of bone. Patients in the control group were treated with symptomatic treatment and functional training of continuous passive motion (CPM), among which, 22 cases were males and 8 cases were females, aged from 15 to 33 years (average (23.7±7.8) years). In the control group, there were 12 cases of distal femoral osteosarcoma, 8 cases of giant cell tumor of bone, 9 cases of proximal tibia osteosarcoma, one case of Ewing’s sarcoma and four cases of giant cell tumor of bone; differences of sex, age, and pathogenic site between two groups of patients were not statistically significant (P>0.05); hence the results were comparable.

### Methods

### Functional training method

Patients in the control group were treated with CPM, and the specific way was as follows. CPM started three days after operation. Patients were required to bend knees starting from 30°, with an increase of 10° each day, 2-3 times a day, one hour once time. The cycle of bending and stretching was about 45s, and the activity lasted for two weeks.

Patients in the observation group were treated with comprehensive rehabilitation, and the specific way was as follows. First was preoperative preparation. General conditions, cardio-pulmonary functions, and mental and intellectual conditions of patients were evaluated. Patients in the group were mainly young adults, and mental stresses of patients and their relatives were relatively heavy, thus, psychological counseling should be taken into consideration; whether patients had bleeding tendency and history of drug allergy and whether their hips, knees and ankles were partly malformed should be also considered. The second step was preoperative rehabilitation guidance. The intensity of contraction training of quadriceps femoris of affected limbs should be increased. Quadriceps femoris contracted for 5s a time, and took a break for 10s. 10 times were regarded as group, and it was required to do 5 to 10 groups a day; the active contraction of flexors and extensors of ankle joints and the functional training of limbs of healthy sides were also done. When knees pained significantly, methods such as preoperative analgesia could be offered to relieve pains. Patients were instructed to move by using walking aids, which was a preparation for postoperative rehabilitation. The third step was rehabilitation training. From 6 hours after operation to the next day, the patients were asked to do ankle pump exercises, namely, bending, stretching and rotation of ankles. Three to five days after operation, patients were required to bend and stretch ankles on their own for 15 minutes each time, with an interval of 2-3 hours, for four times. CPM in an angle of 0° to 30° was performed for 30 min, once a day. Six to eight days after operations, patients should practice embracing and lifting thighs as well as bending knee on their own, 15 minutes each time, every 2 hours. The patients were asked to lie supinely beside beds, and their shanks of affected sides hung beside the edge of beds. Then they exercised for 10 minutes every two hours, and conducted CPM in an angle of 0° to 60° for 30 minutes, twice a day. Nine to twelve days after operation, two legs hung beside beds. The healthy sides would help the affected limbs do uplifting or pushing, for 15 minutes each time, once every two hours. CPM in an angle of 0° to 90° was performed for 45 minutes, twice a day. Two weeks after operation, the patients walked with loads with the help of walking aid and held rails to do squatting (20 times as one group, for three to four groups everyday). The last step was perioperative period combined with (MMA). MMA meant to achieve analgesia by using one or more analgesic drugs or methods.

### Mode of analgesia

Patients in the observation group were treated with MMA, and analgesic drugs and methods were determined according to the specific condition of every patient. For patients who suffered from intensive pain before operations, preemptive analgesia was adopted. Various analgesic methods, such as femoral nerve block, analgesia pumps, partial cold applications and psychological intervention, were adopted after operation. Patients in the control group were only given drugs according to pain before and after surgery.

### Observational index

(1) The training compliance of patients in two groups was observed. (2) The first-time off-bed time of patients in two groups were observed. (3) Recovery conditions of wounds after operations of patients of two groups were observed. Wound healing was divided into three grades: grade A: heal well, with no adverse reactions; grade B: heal with inflammations, such as swelling, induration, hematoma, hydrops and etc., but there was no fester; grade C: fester which required incision and drainage. (4) Hospital for special surgery knee (HSS) score was adopted to evaluate functions of knees, and range of motion (ROM) was measured by ordinary goniometers. The evaluation of knees and measurement of ROM of patients in the two groups were performed in the 2nd week, 3rd month, and 6th month after operations, respectively.

### Statistical analysis

SPSS 21.0 was used to do statistical analysis. Measurement data were expressed by mean ± SD and were tested by using t-test, while the enumeration data were processed by Chi-square test. If P<0.05, difference was considered to be statistically significant.

## RESULTS

### Comparisons of compliance between two groups

Results showed that, the compliance of the observation group was obviously higher than that of the control group. The proportion of patients showing complete compliance was 83.3% in the observation group, which was obviously higher than that of the control group (53.3%), and the difference was statistically significant (P<0.05). Table-I.

**Table-I:** Comparison of compliance between two groups [N(%)].

*Group*	*Complete compliance*	*Partial compliance*	*Non-compliance*
Observation group(N=30)	25(83.3)	3(10.0)	2(6.7)
Control group(N=30)	16(53.3)	8(26.7)	6(20.0)
X^2^	5. 286	1. 224	0.748
P	<0. 05	>0.05	>0.05

### Comparison of the first-time off-bed time between two groups

**Fig.1:**
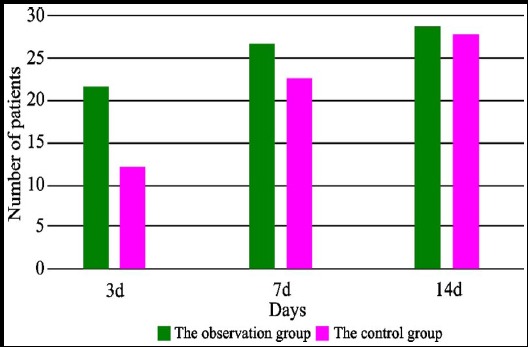
The number of patients who went off from bed for the first time from the 3^rd^ day to 14^th^ day after operations

Results showed that, the difference of the number of people who went off bed for the first time at 3rd day after operation between two groups was statistically significant (P<0.05); the difference at 7th day after operation was statistically significant (P<0.05); the difference at 14 days after operations was not statistically significant (P>0.05) (Fig.1).

### Comparison of recovery conditions of postoperative wounds between two groups

The distribution difference of grade A wound healing at 7th day after operation between the two groups was significant (P<0.05), and the difference 2 weeks after operation was not statistically significant (P>0.05) (Table-II).

**Table-II:** Comparison of the number of cases of grade A healing between two groups 7 days and 2 weeks after operation.

*Group*	*7 days after operation*	*2 weeks after operation*
Observation group(N=30)	22(73.3)	27(90.0)
Control group(N=30)	17(56.7)	26(86.7)
X^2^	4.379	2.016
P	<0. 05	>0.05

### Comparison of HSS score and ROM of knees between two groups

In the comparison of HSS scores and ROM of knees two weeks, three months and 6 months after operation between two groups, the differences were statistically significant (P<0.05). In the comparison of HSS scores 2 weeks and three months after operation, P<0.01; in the comparison of HSS scores 6 months after operation, P<0.05; in the comparison of ROM of knees 2 weeks after operation, P<0.01; in the comparison of HSS scores three and six months after operations, P<0.05. It indicated that, comprehensive rehabilitation training combined with MMA could accelerate the recovery of knee joint functions of patients with bone tumors in knee who underwent prosthesis replacement. Table-III.

**Table-III:** Comparison of HSS score and ROM of knees between two groups (mean ± SD).

*Group*	*2 weeks after operations*		*3 months after operations*		*6 months after operations*	

	*HSS score (point)*	*ROM (degree)*	*HSS score (point)*	*ROM (degree)*	*HSS score (point)*	*ROM (degree)*
Observation group (N=30)	72.39±7.71*	81.18±7.41*	80.62±6.56*	90.74±7.62^#^	87.73±8.29^#^	95.36±6.22^#^
Control group (N=30)	34.62±6.53	59.25±6.57	50.37±6.11	70.32±6.47	71.04±4.28	79.81±6.44

* *Note:* means P<0. 01 and

# means P<0. 05, compared with the control group.

## DISCUSSION

The Incidence of primary bone tumors in knees is relatively high among adolescents; currently the application of customized knee prosthesis remarkably reduces disability rate, especially for adolescents. The applications of prosthesis greatly improves the life quality of patients and extends their lifetime.^10^ With the extensive use of chemotherapy and customized knee replacement operation, people tend to pay more and more attention to the application of active recovery treatment after joint replacement operation. Early rehabilitation training can effectively improve bending functions of affected knees in a short time, alleviate joint swelling, relieve pains and help ROM of knees to reach desired ranges much earlier.^11^,^12^ Postoperative pain is an important factor that limits early rehabilitation training.^13^ Preoperative educations and guidance can be adopted to help patients make enough preparation for postoperative pain, and new concepts and methods of multimodal analgesia should be taken full advantages of, in order to relieve pains after operation and make early rehabilitation training possible under the premise of analgesia.^14^,^15^ Multimodal analgesia aims at effective analgesia. In this study, patients used analgesia pumps after operation, among which 16 patients were treated with femoral nerve block analgesia. Femoral nerve block is an analgesia which can make up for the inadequacy of analgesic drugs used for the whole body.^16^ By means of multimodal analgesia, the compliance of patients of the observation group significantly increased, and they did rehabilitation training without pains, which not only made the recovery of functions quicker and more effective, but also obviously extended the lifetime of patients under a good state of mind.^17^,^18^

Results of the study showed, the number of patients in the observation group that went off from bed for the first time at the 3th day and 7th day was much more than that of the control group, and the difference was statistically significant (P<0.05). It indicates that, with the development and advancement of science and technology, most patients can walk 7 days after operation, but applying the treatment mode of comprehensive rehabilitation training combined with multimodal analgesia can help patients to recover functions of knees and walk earlier after operation. In addition, the number of cases of grade A wound healing of the observation group 7 days after operation was more than that of the control group, and the difference was statistically significant (P<0.05), which indicated that therapeutic scheme of rehabilitation training could promote the healing of wounds more effectively. Moreover, HSS score and ROM of knees of the observation group after operation were both better than those of the control group, which indicates that the efficacy of treatment mode of comprehensive rehabilitation training combined with multimodal analgesia is significant on postoperative recovery of patients with bone tumor on knee who undergo knee prosthesis replacement.

Due to heavy invasion of knee joint prosthesis replacement and little coverage of local software, too early or intense exercise may induce subcutaneous hemorrhage and infection. To avoid the failure of limb salvage treatment, exercise for patients undergoing knee joint prosthesis replacement should be later than exercise for patients undergoing surface knee joint replacement. In this study, the patients of the observation group were treated by local cold compress and only did ankle pump exercise and quadriceps femoris contraction exercise within two days after surgery and did CPM functional training in the 3^th^ to 5^th^ day. The research results suggested such a rehabilitation training mode had remarkable effect.

## CONCLUSION

In the treatment of patients who suffer from bone tumor on knees and underwent tumor bone resection and customized prosthesis replacement, comprehensive rehabilitation training combined with MMA can effectively alleviate negative emotions of patients, help patients to build confidence, enhance the enthusiasm of patients to do functional training after operation, and improve their life quality.
